# Overexpression of plasma membrane SUT1 in poplar alters lateral sucrose partitioning in stem and promotes leaf necrosis

**DOI:** 10.1002/pld3.70023

**Published:** 2025-03-12

**Authors:** Liang‐Jiao Xue, Moh'd I. Hozain, Christopher J. Frost, Afraz Talebi, Batbayar Nyamdari, Kavita B. Aulakh, Ran Zhou, Scott A. Harding, Chung‐Jui Tsai

**Affiliations:** ^1^ Warnell School of Forestry and Natural Resources University of Georgia Athens GA USA; ^2^ Department of Genetics University of Georgia Athens GA USA; ^3^ State Key Laboratory of Tree Genetics and Breeding, College of Forestry Nanjing Forestry University Nanjing Jiangsu China; ^4^ BIO5 Institute Danville VA USA; ^5^ Department of Plant Biology University of Georgia Athens GA USA

## Abstract

In *Populus* and many other tree species, photoassimilate sucrose diffuses down a concentration gradient via symplastically connected mesophyll cells to minor vein phloem for long‐distance transport. There is no evidence for apoplastic phloem‐loading in *Populus*. However, plasma membrane sucrose transporters (SUT1 and SUT3) orthologous to those associated with apoplastic phloem loading are expressed in vascular tissues of poplar. While SUT3 functions in sucrose import into developing xylem, the role of SUT1 remains unclear. Here, we overexpressed *PtaSUT1* in *Populus tremula* x *P. alba* to examine the effects on sucrose partitioning in transgenic plants. Overall leaf sucrose levels were similar between wild type and transgenic lines. Stem sucrose levels were not changed in bark but were significantly reduced in the adjacent xylem, suggesting hindered intercellular sucrose trafficking from the phloem to the developing xylem. Fully expanded leaves of transgenic plants deteriorated prematurely with declining photosynthesis prior to severe necrotic spotting. Necrotic spotting advanced most rapidly in the distal portion of mature leaves and was accompanied by sharp hexose increases and sharp sucrose decreases there. Leaf transcriptome profiling and network inference revealed the down‐regulation of copper proteins and elevated expression of copper microRNAs prior to noticeable leaf injury. Our results suggest ectopic expression of *PtaSUT1* altered sucrose partitioning in stems with systemic effects on leaf health and copper homeostasis mediated in part by sucrose‐sensitive copper miRNAs.

## 1 INTRODUCTION

The long‐distance transport of sucrose (Suc) from source to sink organs is driven by apoplastic or symplastic phloem loading, depending on the species (Rennie & Turgeon, 2009). In apoplastic phloem loading species, plasma membrane‐localized Suc efflux proteins (SWEETs) and proton‐coupled Suc transporters (SUTs) cooperate to mediate the export of Suc from phloem parenchyma into the apoplast and then into the sieve element–companion cell complex of the phloem (Baker et al., 2012; Chen et al., 2012). Apoplastic phloem loading depends on Type I SUTs in dicots and Type II SUTs in monocots (see Peng et al., 2014) and mutations of these SUTs severely impair leaf function and plant growth in *Arabidopsis* and maize (Gottwald et al., 2000; Slewinski et al., 2009). In many tree species including *Populus*, Suc concentrations are high in mesophyll cells of source leaves, and phloem loading depends on passive symplastic diffusion of Suc (Fu et al., 2011; Rennie & Turgeon, 2009; Zhang et al., 2014). However, as a general rule, transport sucrose continually leaks into the apoplast and is recovered by the action of plasma membrane SUTs (Srivastava et al., 2008). The gene encoding Type I PtaSUT3 is weakly expressed in leaves of *Populus tremula* × *alba* and specifically localized to vascular bundles (Payyavula et al., 2011; Xue et al., 2016). *PtaSUT3* is strongly expressed in stems undergoing secondary cell wall thickening (Payyavula et al., 2011). Tracer studies and RNAi‐silencing of its ortholog in *P. tremula* × *tremuloides* demonstrated the role of SUT3 in mediating Suc uptake by fiber‐forming cells (Mahboubi et al., 2013). Whether PtaSUT3 or its genome duplicate PtaSUT1 has a role in fine‐tuning apoplastic Suc levels for additional purposes is not known.

Suc and its component sugars also play important roles in plant interactions with herbivores, pathogens, and beneficial mycorrhizae and endophytes (An et al., 2019; Bitterlich et al., 2014; Breia et al., 2021; Chen et al., 2023; Liu et al., 2022). Pathogens and beneficial endophytes alike can access plant carbohydrates, obviously with contrasting outcomes for the host (Gayosso‐Rosales et al., 2023; Herbers et al., 1996). Pathogen infection of grapevine is known to activate cell wall invertases (CWIs) which hydrolyze apoplastic Suc to hexoses for the benefit of the pathogen (Hayes et al., 2010). The pathogenic bacteria *Xanthomonas* spp. secrete transcription activator‐like effectors into the apoplast to promote virulence (Boch et al., 2014), for example by activating clade III SWEETs to increase Suc efflux into the apoplast (Chen et al., 2010; Streubel et al., 2013). On the other hand, infected plants deploy a range of counter‐defenses, including upregulation of plasma membrane‐localized sugar (hexose) transporters (STPs, also known as HTs) for retrieval of hexoses from the apoplast and induction of pathogenesis‐related proteins (PRs) and secondary metabolite biosynthesis to limit bacterial growth (Hayes et al., 2010; Herbers et al., 1996; Yamada et al., 2016). The hijacking of host sugars by pathogens and the defense‐associated hexose uptake into the cytosol effectively result in the source‐to‐sink transition of infected leaves, and, along with reduced photosynthesis, alter carbon availability (Hayes et al., 2010; Herbers et al., 1996).

*Populus* leaves exhibit conditional sensitivity to perturbations in apoplastic Suc homeostasis caused by CWI. For instance, transgenic *Populus* expressing a yeast‐derived CWI exhibits injury symptoms attributable to apoplastic hexose, but only under conditions of severe abiotic stress (Zhang et al., 2014). By contrast, similarly engineered apoplastic loader alfalfa (*Medicago sativa*) developed severe symptoms consistent with CWI‐mediated hydrolysis of apoplastic Suc even under ambient conditions (Zhang et al., 2014). Therefore, symplastic phloem loaders may be in general less sensitive than apoplastic phloem loaders to perturbations in apoplastic‐symplastic sugar trafficking.

Compared to SWEET and CWI, there are far fewer reported cases of SUT hijacking by pathogens since plasma membrane SUTs are more associated with Suc uptake from the apoplast (Chen et al., 2023). Rather than hijacking SUT, pathogenic fungi like the corn smut *Ustilago maydis* express their own high affinity SUT in order to outcompete the host SUT and CWI for apoplastic Suc (Wahl et al., 2010). In view of the potential competition by beneficial endophytes and pathogens (Gayosso‐Rosales et al., 2023; Suryanarayanan et al., 2022) and the importance of apoplastic sugars to symplastically isolated tissues (Kebrom & Doust, 2022), an intercellular balance must be maintained in which consequences for the host can be positive or deleterious depending on the over‐ or under‐supply of apoplastic sugars. Here we show that constitutive expression of Type I *PtaSUT1* in *P. tremula* × *P. alba* altered sugar partitioning in the stem and yielded a leaf phenotype consistent with defense activation under nonstress conditions. Leaf transcriptome profiling revealed a defense response with a possible connection to sugar and copper (Cu) homeostasis.

## 2 RESULTS

### 2.1 *SUT1* overexpression induced leaf necrosis, decreased photosynthesis, and accelerated senescence

**FIGURE 1:**
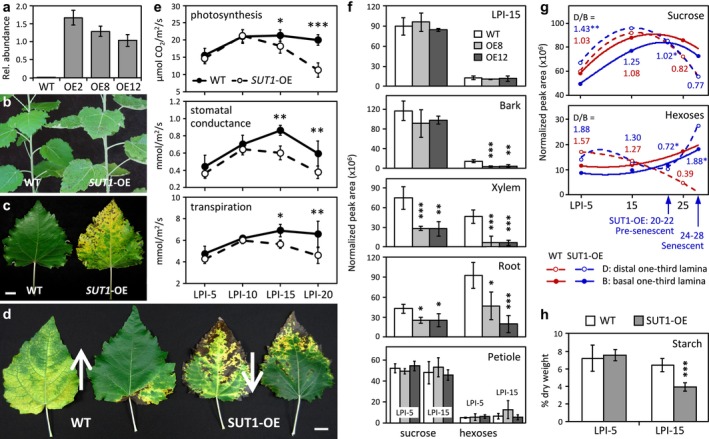
Overexpression of *PtaSUT1* caused leaf necrosis, premature senescence, decreased photosynthesis, and altered sugar partitioning. **a.** *PtaSUT1* transcript abundance in leaves of WT and *SUT1*‐OE plants determined by qRT‐PCR. Values represent means ± SD of *n* = 3 biological replicates. **b.** Necrotic spots in *SUT1*‐OE leaves. **c.** Premature leaf senescence of a representative *SUT1*‐OE leaf. Scale bar = 2 cm. **d.** Acropetal and basipetal progression (arrows) of leaf senescence in WT and *SUT1*‐OE leaves, respectively. Scale bar = 2 cm. **e.** Photosynthesis, stomatal conductance, and transpiration rates of WT and *SUT1*‐OE leaves. Values represent means ± SD of *n* = 3–4 WT or *n* = 4 *SUT1*‐OE (pooled from OE8 and OE12) biological replicates. Statistical significance between WT and *SUT1*‐OE was determined by the two‐sample *t*‐test (*** *P* ≤ .001, ** .001 < *P* ≤ .01, and * .01 < *P* ≤ .05). **f.** Relative sucrose and hexose levels in source (LPI‐15) leaf, bark, xylem, root, and petiole tissues. Values represent means ± SD of *n* = 3–4 biological replicates. **g.** Changes in sucrose and hexose concentrations during leaf development. The average data were applied for curve fitting using quadratic polynomial regression. The distal/basal (D/B) ratios were calculated from *n* = 3–4 WT or *n* = 5–8 *SUT1*‐OE biological replicates. Statistical significance between WT and *SUT1*‐OE was determined using the two‐sample *t*‐test (*** *P* ≤ .001, ** .001 < *P* ≤ .01, and * .01 < *P* ≤ .05). **h.** Starch contents in young (LPI‐5) and mature (LPI‐15) leaves. Values represent means ± SD of *n* = 4 WT or *n* = 5–6 *SUT1*‐OE biological replicates.

Type I *PtaSUT1* and *PtaSUT3* are genome duplicates with a 93% sequence similarity at the amino acid level (Payyavula et al., 2011; Zhou et al., 2023). To avoid potential cosuppression, we targeted *PtaSUT1* normally absent in *Populus* leaves for overexpression in *P. tremula* × *alba* (INRA 717‐1B4) under the control of the 35S promoter. qRT‐PCR screening of putative transformants identified three transgenic lines with strong ectopic *SUT1* expression in leaves and the overexpression was further confirmed in vegetatively propagated plants (Figure 1a). Hereafter, these plants are referred to as *SUT1‐*OE lines. We also observed strong ectopic *SUT1* expression in stem bark and xylem of *SUT1*‐OE plants, but the expression of the paralogous *PtaSUT3* normally high in stems was not affected (Figure S1A). Plant growth of *SUT1*‐OE lines appeared normal until plants reached a height of 75–100 cm, at which time mature leaves began to show spontaneous necrotic spotting (Figure 1b). The symptoms progressed into premature leaf senescence (Figure 1c) and abscission, which resulted in overall growth reduction. Leaf senescence is not typically observed in WT plants grown under normal greenhouse conditions, but occasionally occurs among partially shaded leaves near the base of larger plants. When observed, leaf senescence in WT progressed acropetally (Figure 1d). In contrast, premature senescence of *SUT1*‐OE plants initiated in the distal portion of the leaf (Figure 1c‐d).

Photosynthetic properties were similar between WT and *SUT1*‐OE plants in young source leaves (leaf plastochron index LPI‐5 and LPI‐10) (Figure 1e). However, *SUT1*‐OE lines exhibited a significant decline in net photosynthesis, stomatal conductance, and transpiration rates in source leaves as early as LPI‐15, before any visible sign of necrosis (Figure 1e). As decreased photosynthesis is known to precede leaf senescence (Quirino et al., 2000), the premature decline in photosynthesis of *SUT1*‐OE lines is consistent with the early onset of leaf senescence in these plants.

### 2.2 Developmental and basal‐to‐distal lamina sugar distributions were altered in transgenic plants

Suc and hexose levels were not changed in whole leaf lamina (LPI‐15) or petioles of *SUT1*‐OE plants (Figure 1f). Suc levels also did not change significantly in the bark but were significantly reduced in the xylem and roots of *SUT1*‐OE plants (Figure 1f). In contrast to the situation in leaves, hexose levels were sharply (80%) lower in transgenic bark and xylem, with lesser though still significant reductions in roots (Figure 1f). The bark hexose reductions were especially noteworthy since Suc abundance was not significantly changed there.

Interestingly, Suc levels trended higher in the distal one‐third and lower in the basal one‐third of leaf lamina in *SUT1*‐OE plants relative to WT, especially during early expansion (Figure 1g). As a result, the distal‐to‐basal ratio (D/B) of Suc was significantly higher in LPI‐5 of *SUT1*‐OE than in WT, and decreased with leaf age in *SUT1*‐OE, but not WT, plants (Figure 1g). Hexose levels in basal portions increased gradually with age in both WT and *SUT1*‐OE plants, and like Suc, were slightly lower in *SUT1*‐OE plants (Figure 1g). Distal hexose levels exhibited a steady decline as leaves matured in WT, consistent with whole‐leaf decreases we have reported for poplar in the past (Jeong et al., 2004). However, hexose levels in the distal portion of *SUT1*‐OE leaves decreased only slightly from LPI‐5 to pre‐senescent leaves and then increased markedly in senescing leaves (Figure 1g). Starch levels were not changed in sink leaves but were significantly reduced in LPI‐15 of *SUT1*‐OE plants (Figure 1h).

### 2.3 RNA‐seq analysis revealed altered sugar and copper homeostasis in *SUT1*‐OE leaves

**FIGURE 2:**
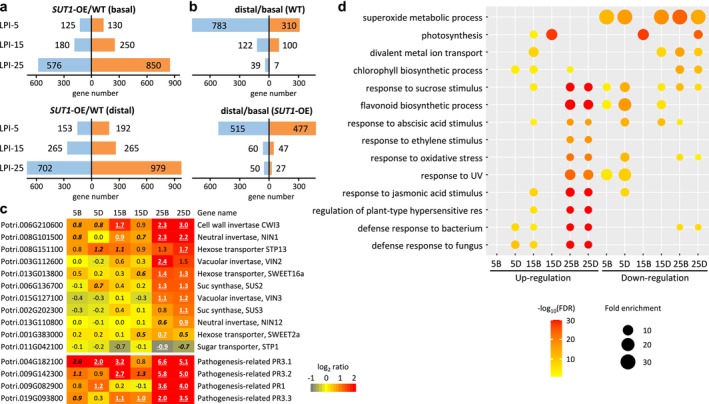
Leaf transcriptional responses in *SUT1*‐OE poplars. **a.** Numbers of significantly up‐ (orange) or down‐regulated (blue) genes in *SUT1*‐OE versus WT plants for basal (top panel) and distal (lower panel) portions of lamina at each indicated leaf age. **b.** Numbers of significantly up‐ (orange) or down‐regulated (blue) genes in distal versus basal lamina at each indicated leaf age of WT (top panel) and *SUT1*‐OE (bottom) plants. Significant differences were based on *P* ≤ .005 and fold‐change ≥ 1.5, with *n* = 3 biological replicates for WT (except for LPI‐25B with *n* = 2) and *n* = 5–6 for *SUT1*‐OE (pooled from *SUT1*‐OE8 and OE12). **c.** Heatmap depiction of transgenic expression responses of genes encoding sucrose cleavage enzymes, sugar transporters, and pathogenesis‐related proteins. Values are log2‐transformed expression ratios (bold‐underlined, *P* ≤ .005; bold‐italics, *P* ≤ .05). **d.** GO functional enrichment analysis of up‐ or down‐regulated genes in *SUT1*‐OE versus WT plants at each indicated leaf age (LPI) and distal (D) or basal (B) lamina portion.

To investigate the transcriptional basis for the strong *SUT1*‐OE leaf phenotypes, RNA‐Seq was performed using basal (B) and distal (D) lamina at three developmental stages. We focused on young, pre‐symptomatic source (LPI‐5 and LPI‐15) and pre‐senescent (green with necrotic spots) leaves based on the altered photosynthesis and sugar distributions described above. We excluded senescing leaves of *SUT1*‐OE plants due to their deteriorated condition. The pre‐senescent leaves of *SUT1*‐OE were collected from LPI‐20 to LPI‐22, a few positions above senescing leaves (LPI‐24 to LPI‐28, depending on the plant), and were paired with LPI‐25 of WT for differential expression analysis (this pair is referred to as LPI‐25 hereafter for simplicity). Because the two *SUT1*‐OE lines exhibited similar phenotypes, data from the two transgenic lines were combined for comparison with WT. The transcriptional response to ectopic expression of *SUT1* increased with leaf age (Figure 2a). There were 255–345 differentially expressed genes (DEGs, *P* ≤ .005, fold‐change ≥1.5) between WT and *SUT1*‐OE at LPI‐5B and LPI‐5D, and the numbers increased slightly at LPI‐15B and LPI‐15D, to 430–530 (Dataset S1). However, more than three times as many DE genes were detected at LPI‐25B and LPI‐25D (Figure 2a), consistent with marked photosynthesis and carbohydrate changes there. When compared between distal and basal lamina (D/B), expression differences decreased as leaves aged in both WT and *SUT1‐*OE plants (Figure 2b).

We confirmed *SUT1* overexpression in OE8 and OE12 lines across all leaf samples, whereas transcript levels of the other *SUT* genes were not affected (Figure S1B‐C). We then examined genes encoding Suc cleavage and sugar transport proteins that may compete for apoplastic sugars (Yamada et al., 2016). We observed significant up‐regulation of *CWI3* (Potri.006G210600), *NIN1* (Potri.008G101500, encoding cytoplasmic neutral invertase), and *STP13* (Potri.008G151100) across *SUT1*‐OE leaves. Genes encoding other Suc cleavage enzymes (Suc synthases [SUSs], vacuolar invertases [VINs], and NIN12) and hexose transporters (plasma membrane/Golgi SWEET16a, vacuolar SWEET2a, and plasma membrane SPT1) were affected mainly in LPI‐25 (Figure 2c) where hexose levels were altered in *SUT1*‐OE lines.

To further assess transcriptomic changes during leaf development, we grouped DEGs by their transgenic up‐ or down‐regulation for functional enrichment analysis. Gene Ontology (GO) categories of ‘regulation of plant‐type hypersensitive response’, ‘defense response to bacterium’, and ‘defense response to fungus’ were over‐represented among up‐regulated DEGs across leaf stages (Figure 2d). Examples included orthologs of multiple PR and PR‐like genes involved in defense (Figure 2c) (Stintzi et al., 1993; van Loon et al., 2006). The large groups of up‐regulated DEGs in LPI‐25 were enriched with functions associated with responses to sucrose and various hormone (jasmonic acid, abscisic acid, and ethylene) stimuli and ‘flavonoid metabolic process’ (Figure 2d). GO terms related to ‘photosynthesis’, ‘chlorophyll biosynthetic process’, and ‘divalent metal ion transport’ were over‐represented among DEGs down‐regulated in LPI‐25 (Figure 2d).

**FIGURE 3:**
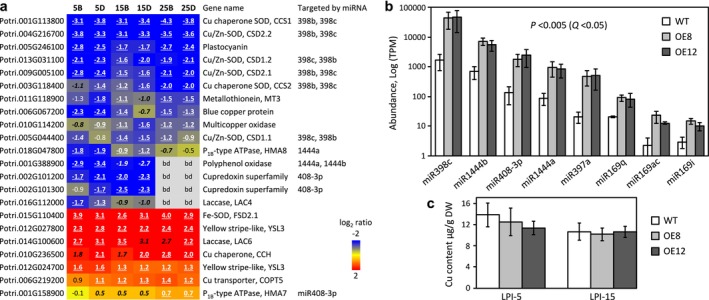
Expression patterns of copper‐sensitive genes and miRNAs. **a.** Heatmap depiction of transgenic effects on genes encoding copper proteins, copper transporters, or otherwise sensitive to copper availability, at the specified leaf (LPI) age and distal (D) or basal (B) lamina portion. The miRNAs predicted to target these genes are shown to the right. Values represent log2‐transformed expression ratios. Significant differences are denoted by bold‐underlined (*P* ≤ .005) or bold‐italics (*P* ≤ .05). bd, below detection. **b.** Differentially expressed miRNAs in LPI‐5D of *SUT1*‐OE plants relative to the WT (*n* = 3). The Y‐axis is log10‐transformed abundance in TPM (transcripts per million reads). **c.** Copper contents of WT and *SUT1*‐OE leaves. Values are means ± SD of *n* = 4–5 biological replicates.

Interestingly, superoxide metabolic process was over‐represented among DEGs down‐regulated in transgenic leaves at all developmental stages (Figure 2d). We observed widespread down‐regulation in *SUT1*‐OE leaves of genes encoding all known Cu/Zn superoxide dismutase (SOD, named CSD) and Cu chaperone for SOD (CCS) isoforms (Molina‐Rueda et al., 2013), as well as plastocyanin, the most abundant Cu protein in poplar leaves (Shahbaz et al., 2015) (Figure 3a). Up‐regulation of genes implicated in Cu transport and translocation (Curie et al., 2009; Pilon, 2011) was also noted, including orthologs of vacuolar Cu transporter *COPT5*, heavy‐metal‐transporting P_1B_‐type ATPase (*HMA7*), Cu chaperone (*CCH*), and yellow stripe‐like metal‐nicotianamine transporters (*YSL3*) (Figure 3a). Down‐regulation of Cu proteins and up‐regulation of Cu transporters are common responses to Cu‐limitation, frequently accompanied by elevated expression of chloroplastic Fe‐SOD (*FSD*) to compensate for *CSD* repression (Pilon et al., 2009) (Figure 3a). These data raised the possibility of altered Cu homeostasis in *SUT1*‐OE plants.

### 2.4 Cu‐sensitive microRNAs were up‐regulated in *SUT1*‐OE plants

Many of the Cu proteins down‐regulated in *SUT1*‐OE plants are known targets of Cu‐miRNAs, such as miR397, miR398, miR408, miR857, and miR1444 (reviewed in Burkhead et al., 2009), some of which are also induced by Suc and/or other abiotic stresses (Dugas & Bartel, 2008; Pilon, 2017). We performed small RNA (sRNA)‐Seq of LPI‐5D to capture the early response of transcriptional reprogramming prior to measurable morphological and physiological changes. Eight annotated miRNA families showed significant differences in both *SUT1*‐OE lines relative to WT, and all were up‐regulated (Figure 3b, Dataset S2). Five of them are Cu‐miRNAs (miR398c, miR1444a/b, miR408, and miR397a), while three belong to the miR169 family (miR169ac, miR169i, and miR169q) that have also been implicated in Suc stress response (Azad et al., 2023). Of these, miR398c constituted 4.5% of total sRNAs in *SUT1*‐OE lines versus only .2% in WT. The response of Cu‐miRNAs prompted us to measure foliar Cu levels. We found no significant difference in the Cu content of either LPI‐5 or LPI‐15 between WT and transgenic plants (Figure 3c). While we cannot exclude the possibility of altered subcellular Cu compartmentalization (see Printz et al., 2016) in the *SUT1*‐OE lines, altered sugar partitioning might have caused the miRNA induction in the transgenics.

### 2.5 Gene co‐expression network rewiring in *SUT1*‐OE leaves

**FIGURE 4:**
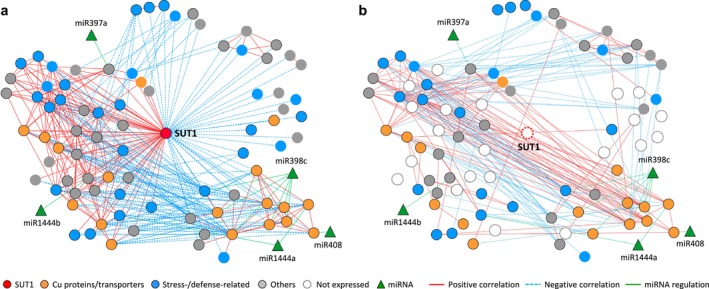
Ectopic *SUT1*‐mediated gene coexpression network rewiring. **a‐b.** Network visualization of *SUT1*‐coexpressed genes inferred from all (WT and *SUT1*‐OE) **(a)** or WT‐only **(b)** samples. Red and blue edges denote positive and negative correlations with *SUT1* (|GCC| ≥ .6), respectively. Genes involved in copper homeostasis and stress responses are colored in orange and blue, respectively, and others in gray. Genes that were below detection in WT leaves are shown as open nodes in **(b)**. miRNAs are shown in green triangles and green edges indicate predicted miRNA regulation. Nodes with solid black outline indicate differential expression between WT and *SUT1*‐OE plants.

A gene co‐expression network incorporating miRNA‐mRNA regulation was constructed using genes that were strongly co‐expressed with *SUT1* (Gini correlation coefficient ≥.6 [44 genes] or ≤ − .6 [38 genes], Dataset S1), along with their predicted recognition by five differentially regulated Cu‐miRNAs for cytoscape visualization (Figure 4). The majority (74%) of the *SUT1*‐coexpressed genes were also DEGs between WT and *SUT1*‐OE lines, enriched with Cu proteins/transporters (orange nodes, Figure 4a), or stress−/defense‐related genes (blue nodes). The observed co‐expression patterns differed markedly when only WT samples were used in the network inference (Figure 4b, genes not expressed in WT samples are shown as white nodes).

The *SUT1*‐OE network was extensive and impinged upon genes involved in Cu homeostasis (*COPT5*, *YSL*, *CCH*, *FSD*, and *plastocyanin*) and defense. The latter included orthologs of the *Arabidopsis* antifungal chitinase (also known as PR3, Attallah et al., 2011), antimicrobial heat‐stable protein 1 (Park et al., 2007), sigma factor binding protein (SIB) known to act upstream of defense‐related WRKY transcription factors (Lai et al., 2011), and an ethylene‐responsive factor (HRE2) involved in hypoxia and osmotic stress response (Park et al., 2011). Several *Populus* genes encoding wound‐inducible bark storage proteins of the nucleoside phosphorylase family (Pettengill et al., 2013) were also captured (Dataset S1).

Genes negatively co‐expressed with *SUT1* consisted predominantly of Cu proteins, many of them predicted targets of Cu‐miRNAs (Figure 4a, Dataset S1). Also included was a NAC transcription factor ortholog (Potri.005G180200) of *Arabidopsis* ATAF1 that is responsive to various biotic and abiotic stresses (Wu et al., 2009) and promotes senescence by regulating ABA homeostasis (Garapati et al., 2015; Jensen et al., 2013). Overall, the *SUT1*‐centered network inference supports a transgenic defense response that was integrated with Cu homeostasis and miRNA regulation.

### 2.6 Chemical defense was altered in *SUT1*‐OE plants

**FIGURE 5:**
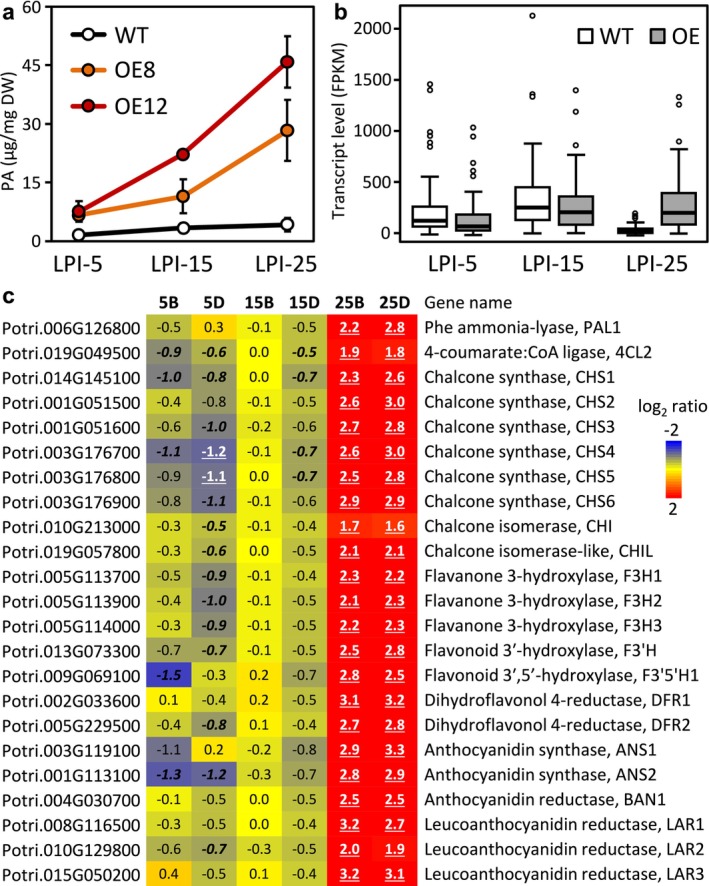
Defense responses of *SUT1*‐OE plants. **a.** PA concentrations in WT and *SUT1*‐OE plants during leaf development. Values are means ± SD of *n* = 4 biological replicates. The differences between WT and each *SUT1*‐OE line were significant (*P* < .005) for all leaf ages. **b‐c.** Box‐plot depiction of PA biosynthetic gene transcript abundance (B). The boxes denote the 25th and 75th percentiles and the medians, the whiskers indicate 1.5 times the interquartile range, and the dots represent outliers (*n* = 46 data points of averaged transcript abundances from WT (*n* = 2–3) or *SUT1*‐OE (*n* = 5–6) samples for 23 PA biosynthetic genes and two (distal and basal) lamina portions at each leaf age shown as heatmap depiction in C. Values represent log2‐transformed expression ratios. Significant differences are denoted by bold‐underlined (*P* ≤ .005) or bold‐italics (*P* ≤ .05).

Salicinoids (phenolic glycosides) and flavonoid‐derived proanthocyanidins (PAs, also called condensed tannins) comprise two quantitatively significant chemical defense pools in *Populus* leaves (Tsai et al., 2006). PAs are of particular relevance, because PAs have been implicated in poplar defense against leaf rust fungi (Miranda et al., 2007; Ullah et al., 2017; Ullah et al., 2022; Yuan et al., 2012), and because PAs are thought to undergo oxidative polymerization in the apoplast for deposition to cell walls (Zhao et al., 2010). We found no changes in salicinoid levels, but PA levels increased significantly in all three stages of *SUT1*‐OE leaves, by 4‐ to 11‐fold (Figure 5a). Interestingly, expression of PA biosynthetic genes was up‐regulated only in older leaves (LPI‐25) where WT transcript levels normally declined (Figure 5b‐c). This suggests that the PA increases in LPI‐5 and LPI‐15 with active flavonoid gene expression may be driven by redirection of carbon flow, whereas elevated transcription was necessary to sustain the high levels of PA in LPI‐25. Regardless, increased PA accruals in transgenic leaves are consistent with an activated defense response in *SUT1*‐OE poplars.

## 3 DISCUSSION

*Populus* species use symplastic phloem loading for long‐distance Suc transport (Zhang et al., 2014). Interestingly, poplar leaves exhibit weak expression of Type I *PtaSUT* orthologous to those involved in apoplastic phloem loading of many annual species (Payyavula et al., 2011; Xue et al., 2016). *PtaSUT3* is most highly expressed in xylem and bark, but its genome duplicate *PtaSUT1* is only detected in bark. Here, we show that ectopic expression of *PtaSUT1* in transgenic *Populus* perturbed Suc partitioning in stems and triggered leaf injury mediated in part by altered Cu homeostasis via Suc‐sensitive Cu‐miRNAs. Activated defense responses led to the sequestration of carbon in the form of PAs and premature leaf senescence. Our data shows that misregulation of *SUT1* in stems could affect the symplastic‐apoplastic dynamic in *Populus*, perhaps with severe consequences in leaves.

Our findings that Suc levels were reduced in the xylem and roots but not in the bark of *SUT1*‐OE stems suggest a role for Type I SUT downstream of phloem transport but upstream of Suc distribution to lateral and terminal sinks. Previous fluorescent tracer studies show clear symplastic continuity between phloem and lateral sinks in woody plants but also reveal regular symplastic discontinuities between fusiform cells and developing xylem (Sokołowska & Zagórska‐Marek, 2012). Indeed in *Populus* stems, Suc unloading and delivery to lateral sinks like wood‐forming tissues is facilitated by plasma membrane SWEET exporters and CWIs, with SUT3 mediating Suc uptake across the plasma membrane by developing xylem fiber cells (Mahboubi et al., 2013; Zhang et al., 2020). The present findings that hexose levels were sharply reduced in both the xylem and bark of *SUT1‐*OE lines are consistent with an involvement of the ectopic SUT1 in this process as well.

While the stem data seem to support the idea that *SUT1*‐OE could have reduced apoplastic Suc, it is not clear if the same process in leaves promoted hypersensitive response‐like symptoms normally associated with the breakdown of apoplastic Suc (Herbers et al., 1996; Zhang et al., 2014). Clade III plasma membrane SWEETs are normally well‐expressed and required for cellular Suc efflux into the apoplast in leaves of annual apoplastic phloem loaders are below detection in *Populus* leaves (Zhang et al., 2020). If PtaSUT1 functions analogously to its paralog PtaSUT3, it is possible that its transgenic overexpression in leaves or bark would increase cellular uptake of Suc and H^+^. Our evidence that this may have occurred consists of the Suc‐hexose perturbations in *SUT1*‐OE stems (Figure 1f) noted above, and of significant upregulation of genes involved in intracellular Suc cleavage (SUSs, NINs, VINs) and hexose trafficking (SWEET2a and SWEET16a) (Figure 2c) that preceded the sharp hexose increases in senescing *SUT1*‐OE leaves (Figure 1g). SUT1‐mediated Suc/H^+^ uptake would cause cytosolic acidification and apoplastic alkalization. Cytosolic acidification is known to act as a second messenger to activate defense signaling whereas apoplastic alkalization has been proposed as a general defense signal in response to biotic and abiotic stimuli, and the signal is transmitted systemically via xylem (Felle et al., 2005; O'Leary et al., 2016). Future research with apoplastic fluid extraction is needed to confirm this possibility. It should be noted that defense activation with leaf necrosis similar to what we observed in *SUT1*‐OE lines was reported for transgenic poplar expressing yeast CWI, especially under extreme heat (Zhang et al., 2014). In the present work, elevated expression of *CWI3*, *STP13*, and *PRs*, increased accumulation of PAs, and decreased photosynthesis along with the appearance of necrotic spots in otherwise healthy source leaves are all consistent with defense responses to altered intra‐ and intercellular sugar trafficking in *SUT1*‐OE leaves.

An unexpected finding was the upregulation of Cu‐miRNAs and the associated downregulation of their predicted targets encoding CSDs, CCSs, and other Cu proteins in *SUT1‐*OE leaves (Figure 3). Cu‐miRNAs are best known for their induction by Cu deficiency, but they are also induced by Suc, hydrogen peroxide, and various other abiotic stimuli (Dugas & Bartel, 2008; Pilon, 2017; Ren & Tang, 2012; Yamasaki et al., 2007). As Cu levels were unchanged in transgenic leaves, the elevated Cu‐miRNA expression in *SUT1*‐OE plants was most likely triggered by altered Suc partitioning. CSDs and CCSs function as reactive oxygen species scavengers. Thus, their widespread down‐regulation had ramifications for redox regulation, leaf necrosis, senescence, and pathogen interactions (Xu et al., 2014). Furthermore, altered expression of several Cu proteins and Cu transporters not known to be regulated by Cu‐miRNAs suggests a secondary effect of ‘perceived Cu deficiency’. Examples include suppression of plastocyanins (Shahbaz et al., 2015) and induction of *YSLs*, *FSD*, and CCH (Curie et al., 2009; Pilon, 2011) in all transgenic leaves. Interestingly, the Arabidopsis ortholog *AtCCH* is highly up‐regulated in senescing leaves and functions in the remobilization of Cu to other growing parts (Himelblau & Amasino, 2000; Mira et al., 2001).

In conclusion, our results suggest that misregulation of plasma membrane SUT proteins altered xylem provisioning with systemic effects on overall growth and leaf health even in species that do not rely on plasma membrane SUTs for the export of photosynthate from source leaves. In addition, the broad‐based molecular evidence supports an alteration of copper homeostasis due to local or systemic Suc perturbations in the *SUT1*‐OE lines mediated by Cu‐miRNAs.

## 4 MATERIAL AND METHODS

### 4.1 Transgenic plant production, plant growth, and tissue collection

The *PtaSUT1* coding sequence in pCRII‐TOPO (Payyavula et al., 2011) was PCR‐amplified, subcloned into *Xcm*I‐digested pCX‐SN (Chen et al., 2009), and sequence‐confirmed before being mobilized into *Agrobacterium tumefaciens* strain C58/pMP90. Leaf disc transformation of *P. tremula* x *alba* (clone 717‐1B4) was performed using an established protocol (Meilan & Ma, 2006). Positive transformants confirmed by PCR were transplanted to soil, along with tissue culture‐propagated WT plants, and grown in a greenhouse. LPI‐5 was used for qRT‐PCR screening of *SUT1* expression, and three independent lines (OE2, OE8, and OE12) with confirmed overexpression were propagated by rooted cuttings and maintained under greenhouse conditions as previously described (Frost et al., 2012). These plants were used for initial characterization and monitoring. LPI‐7 from 3‐month‐old plants were collected for qRT‐PCR confirmation of *SUT1* overexpression using three biological replicates. Independent batches of cuttings from WT, OE8, and OE12 were used for in‐depth characterization. Net photosynthesis, stomatal conductance, and transpiration rates were monitored according to Frost et al. (2012), using a Licor LI‐6400XS at a saturating light intensity of 1,500 μmol/m^2^/s. Plants 1.6–1.8 m in height were destructively harvested to collect LPI‐5, LPI‐15, and LPI‐25 from WT, or LPI‐5, LPI‐15, necrotic pre‐senescent (LPI‐20‐22) and senescent (LPI‐24‐28) leaves from *SUT1*‐OE plants. After collecting the petiole and removing the midrib, each leaf lamina was further divided into distal (D) and basal (B) halves and separately collected. Developing xylem scrapping and bark were obtained from internodes 20–40. Tissues were immediately snap‐frozen in liquid nitrogen and stored at ‐80 °C. Tissues were ground to a fine powder in liquid nitrogen, aliquoted, and stored at ‐80 °C until use.

### 4.2 RNA extraction and qRT‐PCR analysis

Total RNA was extracted from frozen tissue aliquots using the Direct‐zol RNA Kit (Zymo Research) with Plant RNA Reagent (Life Technologies). RNA concentration was determined using a Qubit fluorometer with the Qubit RNA HS Assay Kit (Life Technologies). qRT‐PCR was performed as described (Tsai et al., 2006) using the High‐Capacity cDNA Reverse Transcription Kit (Applied Biosystems) for cDNA synthesis, and the Absolute Blue QPCR mix (Thermo Scientific) for real‐time PCR. Primer information is provided in Table S1.

### 4.3 Chemical analyses

Freeze‐dried tissue powders (10 mg) were used for sugar (Suc, Glc, and Fru) analysis by GC–MS as detailed in Xue et al. (2016). The leaf data were applied for curve fitting using a quadratic polynomial regression to observe spatial patterns during basal‐to‐distal leaf development. The order of the polynomial (2 or 3) was chosen based on the R‐squared value. Another aliquot of LPI‐5B and LPI‐15B powders (10 mg) was used to estimate starch contents by enzymatic digestion as described (Chow & Landhausser, 2004). Total PA levels were assayed using 10 mg of freeze‐dried leaf powders and the colorimetric butanol‐HCl method of Porter et al. (1986). PAs purified from leaves of *P. angustifolia* by LH‐20 chromatography (Strumeyer & Malin, 1975) were used as standards for quantification. For Cu analysis, 50 mg of freeze‐dried leaf powders were ball‐milled and digested along with leaf standards of known metal micronutrient contents in 1 ml of concentrated nitric acid at 95 °C for 3 hr. After cooling, digests were diluted 10‐fold with ddH_2_O and submitted for inductively coupled plasma spectrometry at the Center for Applied Isotope Studies, University of Georgia.

### 4.4 RNA‐Seq analysis

Illumina TruSeq stranded total RNA libraries were prepared according to manufacturer's instructions, and Illumina NextSeq 500 sequencing was performed at the Georgia Genomics and Bioinformatics Core of the University of Georgia. Approximately 6–13 million paired‐end 75‐bp reads were obtained for each sample, with three biological replicates for WT, *SUT1*‐OE8, and *SUT1*‐OE12 except otherwise noted. The read pairs were subjected to quality control filtering and mapped onto the variant‐substituted *P. tremula* x *alba* genome sPta v1.1 as described (Xue et al., 2015) using Tophat2 v2.0.12 (Kim et al., 2013). Transcript abundance was estimated by HTSeq (Anders et al., 2015), and differential expression was determined using DESeq2 (Love et al., 2014) based on *P* ≤ .005, fold‐change ≥1.5, and FPKM ≥3 in all (WT and *SUT1*‐OE) replicates of at least one test group. GO enrichment analysis was performed for DEGs using a local instance of ShinyGO v0.77 (Ge et al., 2020) and *Populus trichocarpa* V3 genome with custom GO annotation from both Phytozome v13 (Goodstein et al., 2012) and *Arabidopsis thaliana* Araport11 best hits (Krishnakumar et al., 2016), available at http://aspendb.uga.edu/ShinyGO/. The enrichment significance was determined by hypergeometric distribution followed by false discovery rate (FDR) correction. The negative log10 transformed (FDR‐adjusted) *P* values and fold enrichment values were visualized in a bubble plot using an in‐house script. Transgenic responses of selected genes were visualized using the HeatMapperPlus tool (http://bar.utoronto.ca/ntools/cgi-bin/ntools_heatmapper_plus.cgi) or BoxPlotR (Spitzer et al., 2014).

### 4.5 Small RNA sequencing and analysis

Illumina TruSeq small RNA libraries were prepared using total RNA from LPI‐5D samples following manufacturer's instructions, and sequenced on a NextSeq 500 at the Georgia Genomics and Bioinformatics Core, University of Georgia. An average of 13 million single‐end reads were obtained per sample (n = 4 for WT, and n = 3 for OE8 and OE12). The reads were trimmed using Cutadapt v1.14 (Martin, 2011), and mapped against rRNA, tRNA, snRNA, and snoRNA databases to unwanted sequences. The retained reads (2.6–5.4 million per sample) were merged to predict miRNA structures and estimate their abundances using ShortStack v3.5 (Johnson et al., 2016). The abundance values of major miRNA species were used to perform differential expression tests using DEseq2 v3.6 (Love et al., 2014). The targets of miRNAs were predicted using psRNATarget (Dai & Zhao, 2011).

### 4.6 Integration of *SUT1*‐coexpression network and miRNA regulatory network

Pairwise Gini Correlation Coefficients (GCC) were calculated across all (WT and *SUT1*‐OE) or WT‐only samples using a Python script (https://github.com/tsailab/PythonCalculation). Genes coexpressed with *SUT1* (|GCC| ≥ .6) were separately extracted from the two networks, and potential miRNA targets were identified based on the psRNATarget prediction described above. The network structures were visualized using Cytoscape v3.2.1 (Shannon et al., 2003), with the organic layout for the all‐sample network. The gene and miRNA connections from the WT network were mapped onto the topology of the all‐sample network for ease of comparison.

### 4.7 Accession numbers

The NCBI Sequence Read Archive (SRA) accession numbers for RNA‐Seq and sRNA‐seq data are SRP123518 and SRP123565, respectively.

## AUTHOR CONTRIBUTIONS

C.‐J.T., S.A.H., and L.‐J.X. designed the research, C.J.F. generated the transgenic plants and performed initial characterization; M.I.H. performed most of the experiments, with technical assistance from A.T., B.N., K.B.A., and S.A.H; L.‐J.X. performed all bioinformatics analyses with help from R.Z.; M.I.H., L.‐J.X., S.A.H., and C.‐J.T. analyzed the data; L.‐J.X. and C.‐J.T. wrote the manuscript with contributions of S.A.H.

## CONFLICT OF INTEREST STATEMENT

The Authors did not report any conflict of interest.

## Supporting information


**Figure S1.** Expression levels of *SUT* genes in transgenic plants.

**Table S1.** Primers.

**Dataset S1.** List of differentially expressed genes based on RNA‐Seq.

**Dataset S2.** List of differentially expressed miRNAs based on sRNA‐Seq.
