# Supplementary material for: Overexpression of plasma membrane SUT1 in poplar alters lateral sucrose partitioning in stem and promotes leaf necrosis
Source: Plant Direct. 2025 Mar 12;9(3):e70023. doi: 10.1002/pld3.70023 (PMC11897725; doi:10.1002/pld3.70023)
Supplement: Supplementary file 1 — Figure S1. Expression levels of SUT genes in transgenic plants. [file PLD3-9-e70023-s003.pdf]

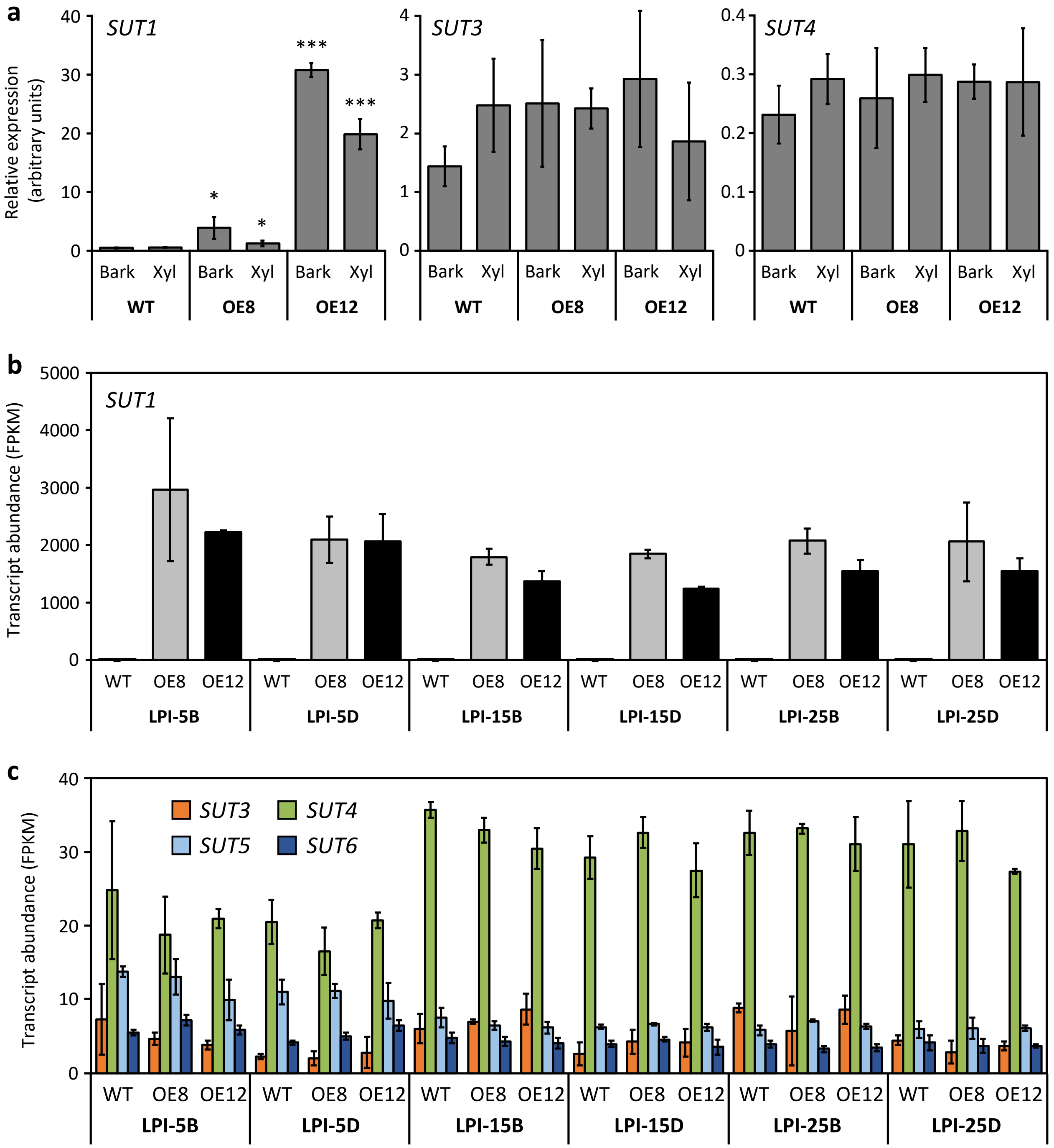

Supplemental Figure 1. Expression levels of *SUT* genes in transgenic plants.
(a) Transcript levels of *SUT1*, *SUT3*, and *SUT4* in bark and xylem were determined by qRT-PCR. Values represent means ± SD of n = 3-4 biological replicates. Significant difference was determined by Student's t-test (*, $P < 0.05$; ***. $P < 0.001$). No significant difference was found between WT and transgenic lines for *SUT3* and *SUT4*. **(b)** Transcript levels of *SUT1* as determined by RNA-Seq (n = 3 biological replicates, except for WT LPI-25B, OE8 LPI-15B and OE12 LPI-5B and LPI-25D where n = 2). The differences between WT and each transgenic line were significant in all cases (P < 4E-38). **(c)** Transcript levels of the other four *SUT* genes as determined by RNA-Seq (replicate numbers are as above). No significant difference was found between WT and transgenic lines.
