# Supplementary material for: Overexpression of plasma membrane SUT1 in poplar alters lateral sucrose partitioning in stem and promotes leaf necrosis
Source: Plant Direct. 2025 Mar 12;9(3):e70023. doi: 10.1002/pld3.70023 (PMC11897725; doi:10.1002/pld3.70023)
Supplement: Supplementary file 2 — Table S1. Primers. [file PLD3-9-e70023-s004.doc]

| **Supplemental Table 1. List of primers** | | | |
| --- | --- | --- | --- |
| Gene | Gene model | 5’-3’ sequence | Purpose |
| SUT 1 | Potri.013G115200 | CATGGAGAGTGGAGTTAGAAAAGAARAC | binary vector cloning |
|  |  | TCAAWGGAATGCARCASWASTACTGGTGGSAGC |  |
|  |  |  |  |
| HPT | n/a | GAGGGCGAAGAATCTCGTGC | PCR confirmation |
|  |  | GATGTTGGCGACCTCGTATTGG |  |
|  |  |  |  |
| SUT1 | Potri.013G115200 | TGGTKTCTGTAGCARSTGGACCTT | qRT-PCR |
|  |  | AACTGCTACCACYACTGCATTCTG |  |
|  |  |  |  |
| SUT3 | Potri.019G085800 | TGGTKTCTGTAGCRRSTGGACCTT | qRT-PCR |
|  |  | ACTAACTGCGGCTGCAACA |  |
|  |  |  |  |
| SUT4 | Potri.002G106900 | ATCCTTGGGACTTGGACAAGGTGG | qRT-PCR |
|  |  | TGATCGACGAATACCCAAGATGGC |  |
|  |  |  |  |
| ACT | Potri.019G010400 | GCGGTGATGGTGAGTTCTTTCT | qRT-PCR control, screening |
|  |  | ATCGAGAGGGAGGACCATTACAGT |  |
|  |  |  |  |
| UBC | Potri.006G205700 | CTGAAGAAGGAGATGACARCMCCA | qRT-PCR control, Fig. 1A |
|  |  | GCATCCCTTCAACACAGTTTCAMG |  |
|  |  |  |  |
| ARP | Potri.017G057400 | ACTGTGAGGAGATGCAGAAACGCA | qRT-PCR control, Fig. S1 |
|  |  | GCTGTGTCACGGGCATTCAATGYT |  |
|  |  |  |  |
| ELF | Potri.001G224700 | GACCTKGTATCAGTGGATTCCCTC | qRT-PCR control, Fig. S1 |
|  |  | GAACAGAGGCACAAGATTACCAGG |  |
